# IGF2BP3 promotes glutamine metabolism of endometriosis by interacting with UCA1 to enhances the mRNA stability of GLS1

**DOI:** 10.1186/s10020-024-00834-7

**Published:** 2024-05-17

**Authors:** Honglin Wang, Yingying Cao, Yanling Gou, Hao Wang, Zongwen Liang, Qiong Wu, Jiahuan Tan, Jinming Liu, Zhi Li, Jing Cui, Huiyan Zhang, Zongfeng Zhang

**Affiliations:** 1 Department of Obstetrics and Gynecology, Second Affiliated Hospital of Harbin Medical University, 148 Baojian Road, Harbin, 150086 China; 2 Department of Obstetrics and Gynecology, Shanghai General Hospital, Shanghai Jiaotong University School of Medicine, No. 100 Haining Road, Hongkou District, Shanghai, 200080 China; 3 Department of Obstetrics and Gynecology, Zhongda Hospital Southeast University (Jiangbei), NanJing, China

**Keywords:** Endometriosis, IGF2BP3, Glutamine metabolism, GLS1, UCA1, c-MYC

## Abstract

**Background:**

Insulin like growth factor II mRNA binding protein 3 (IGF2BP3) has been implicated in numerous inflammatory and cancerous conditions. However, its precise molecular mechanisms in endometriosis (EMs) remains unclear. The aim of this study is to examine the influence of IGF2BP3 on the occurrence and progression of EMs and to elucidate its underlying molecular mechanism.

**Methods:**

Efects of IGF2BP3 on endometriosis were confrmed in vitro and in vivo. Based on bioinformatics analysis, RNA immunoprecipitation (RIP), RNA pull-down assays and Fluorescent in situ hybridization (FISH) were used to show the association between IGF2BP3 and UCA1. Single-cell spatial transcriptomics analysis shows the expression distribution of glutaminase 1 (GLS1) mRNA in EMs. Study the effect on glutamine metabolism after ectopic endometriotic stromal cells (eESCs) were transfected with Sh-IGF2BP3 and Sh-UCA1 lentivirus.

**Results:**

Immunohistochemical staining have revealed that IGF2BP3 was upregulated in ectopic endometriotic lesions (EC) compared to normal endometrial tissues (EN). The proliferation and migration ability of eESCs were greatly reduced by downregulating IGF2BP3. Additionally, IGF2BP3 has been observed to interact with urothelial carcinoma associated 1 (UCA1), leading to increased stability of GLS1 mRNA and subsequently enhancing glutamine metabolism. Results also demonstrated that IGF2BP3 directly interacts with the 3’ UTR region of GLS1 mRNA, influencing its expression and stability. Furthermore, UCA1 was able to bind with c-MYC protein, stabilizing c-MYC mRNA and consequently enhancing GLS1 expression through transcriptional promotion.

**Conclusion:**

These discoveries underscored the critical involvement of IGF2BP3 in the elevation and stability of GLS1 mRNA in the context of glutamine metabolism by interacting with UCA1 in EMs. The implications of our study extended to the identification of possible therapeutic targets for individuals with EMs.

**Supplementary Information:**

The online version contains supplementary material available at 10.1186/s10020-024-00834-7.

## Introduction

Endometriosis (EMs) is a long-term ailment marked by pelvic discomfort and infertility (Giudice 2010; Taylor et al. 2021), impacting approximately 5–10% of women in their childbearing years globally (Simoens et al. 2012). Despite its prevalence, there is a significant lack of awareness surrounding the disease, resulting in prolonged diagnostic journey that can span over a period of 4 to 11 years, with 65% of women receiving misdiagnoses at first (Greene et al. 2009). Furthermore, the standard diagnostic tool, laparoscopy, can be prone to inaccuracies when identifying EMs (Taylor et al. 2021). Hence, a holistic, clinically oriented approach is essential for ensuring precise and timely diagnosis of this condition (Agarwal et al. 2019; Chapron et al. 2019). Diving deeper into the biological and chemical processes of EMs and pinpoingting possible treatment targets are imperative for advancing treatment strategies.

RNA-binding proteins (RBPs) are vital regulators of gene expression and are capable of interacting with numerous transcription factors, forming an extensive regulatory network involved in maintaining cellular homeostasis (Gebauer et al. 2021). Insulin like growth factor II mRNA binding proteins (IGF2BPs) comprises IGF2BP1, IGF2BP2 and IGF2BP3, which are pivotal in disease development through their regulation of mRNA stability and translation. These IGF2BPs modulate key regulatory factors involved in cell division and metabolism, exerting a significant impact on disease progression (Degrauwe et al. 2016; Elcheva et al. 2023). Insulin like growth factor II mRNA binding protein 3 (IGF2BP3), also recognized as IMP3, KOC and VICKZ3, serves as a tumorigenic protein implicated in various cancers (Wan et al. 2022; Yang et al. 2023a, b; Yu et al. 2022). Yang et al. suggested that IGF2BP3 held prognostic value as a marker for ovarian clear cell carcinoma (CCC) (Bi et al. 2016). A significant number of young patients have links to EMs. Previous studies have indicated that the etiology of CCC and EMs encompasses similar pathophysicological and molecular targets, such as the p53 and the Wnt/β-catenin pathways (Chen et al. 2019; Harden et al. 2023; Zhang et al. 2021). However, further investigation needs to elucidate the potential role of the IGF2BPs in the molecular mechanisms underpinning these two conditions.

Extensive metabolic reprogramming and cancer-like changes have been observed in EMs, showing parallels to the process of tumorigenesis (Li et al. 2023; Lu et al. 2022). These include phenomena such as increased aerobic glycolysis (Young et al. 2014), augmented proline biosynthesis, and activated glutaminolysis (Kusum et al. 2022). In the tumour microenvironment (TME), there is an increased demand for glutamine, resulting in heightened activity of glutamine transporters and key enzymes involved in glutamine metabolism. This heightened activity promotes cell survival and proliferation within TME (Chen et al. 2023a,b; Csibi et al. 2013; Li et al. 2019; Liu et al. 2022; Raggi et al. 2022; Zhu et al. 2021). Notably, individuals with EMs exhibit significantly elevated levels of glutamine and reduced levels of tryptophan (Kusum et al. 2022; Murgia et al. 2021). Glutaminase 1 (GLS1) is a crucial enzyme that is instrumental in the initial stage of glutamine metabolism by facilitating the conversion of glutamine to glutamate and ammonia (Yang et al. 2019). Indeed, the specific molecular mechanisms by which the therapeutic intervention of glutamine metabolic pathways and molecules modulates the immune response in EMs are not yet well understood.

Long non-coding RNAs (lncRNAs) are a multiple class of RNA transcripts that usually consists of over 200 nucleotides and have restricted ability to encode proteins (Zhou et al. 2020). The function of IncRNAs is closely associated with their subcellular localization. Within the nucleus, lncRNAs are involved in regulating gene regulation through epigenetic and transcriptional mechanisms. Within the cytoplasm, lncRNAs play a role in regulating gene regulation at the post-transcriptional and translational stages (Xing et al. 2021). Our previous findings suggested that knockdown of lncRNA MALAT1 enhanced the development of ferroptosis caused by erastin treatment. This effect was mediated through the miR-145-5p/MUC1 signaling pathway, which consequently regulated the progression of EMs (Liang et al. 2022). Similarly, Yaling et al. demonstrated that IGF2BP2 facilitated the proliferation, migration and invasion of ectopic endometriotic stromal cells (eESCs) by enhancing the stability of MEIS2 and GATA6 mRNA (Zhao et al. 2022a,b). Nevertheless, our comprehension of how lncRNAs regulate IGF2BP3 in the advancement of EMs remains restricted.

Up to now, the role of IGF2BP3 in EMs has not been comprehensively investigated. In this analysis, we revealed that knockdown of IGF2BP3 in eESCs notably impaired their proliferation, migration and glutamine metabolism capabilities. RBPs can influence cellular function by modulating the activity of lncRNAs or interacting with lncRNAs (Gerstberger et al. 2014). Interestingly, we discovered an interaction between lncRNA urothelial carcinoma associated 1 (UCA1) and IGF2BP3 without mutual regulatory effects. Mechanistically, we discovered that UCA1 enhanced the expression of IGF2BP3 and stabilized GLS1 mRNA. Moreover, UCA1 contributed to the stabilization of c-MYC mRNA and protein abundance, consequently promoting GLS1 expression at the transcriptional step.

In conclusion, our research findings provided insights into the involvement of the IGF2BP3/UCA1/c-MYC/GLS1 signaling axis in the regulation of glutamine metabolism, highlighting its potential as a therapeutic approach for EMs.

## Materials and methods

### Participants and tissue specimens

This study was ethically approved by the Ethics Committee of the Second Affiliated Hospital of Harbin Medical University (Approval Number: KY-2020-030 and YJSKY2022-491). The experimental group comprised 19 female individuals who underwent laparoscopic surgery and were diagnosed with EMs through pathological examination in the gynecology department of our hospital from June 2020 to June 2022. The control group included 16 women without endometritis or adenomyosis, who underwent surgical treatment for other non-cancerous gynecological conditions such as uterine leiomyoma (Table 1). Tissue samples were obtained from patients during the phase of increased cell proliferation in their menstrual cycle. A portion of the tissue was immediately transported to the laboratory for primary cell culture and in vitro experiments using serum-free DMEM medium at 4 ℃. Another portion was soaked in 4% PFA for fixation, and then subjected to paraffin sectioning. The remaining portion was stored at – 80 ℃ for subsequent extraction of RNA or protein. All participants provided informed consent.

**Table 1:** Clinical characteristics of female with and without (control) endometriosis

	Control (n = 16)	Endometriosis (n = 19)	*P* value
Age (year)	40.14 ± 10.36	37.37 ± 7.81	0.349
BMI (Kg/m^2^)	23.58 ± 3.11	23.26 ± 2.96	0.7428
CA125 level (U/mL)	18.68 ± 12.55	64.42 ± 80.15	0.0262
Menstrual average cycle (day)	29.38 ± 1.99	28.32 ± 2.5	0.1418
Menstrual duration (day)	5.33 ± 1.24	5.47 ± 1.54	0.7515
r-AFS stage			
Stage I (minimal)		0/19	
Stage II (mild)		3/19	
Stage III (moderate)		12/19	
Stage IV (severe)		4/19	

### Cell culture

We isolated primary eESCs from 19 patients diagnosed with EMs. Ectopic lesions were obtained from these patients and cut into 1 mm^3^ blocks. These blocks were digested using 4% collagenase type IV (C5138, Sigma, USA) in an oscillating water bath at 37 ℃ for an hour. Separated the endometrial cells by sieves. Centrifuged the filtrate at 1000 × *g* for 5 min. The remaining cells were resuspended and propagated in culture in DMEM supplemented with 15% fetal bovine serum (FBS; Biological Industries, Israel). To confirm the identities of the normal endometrial stromal cells (nESCs) and eESCs, the immunofluorescence staining was performed (Fig. S1). In addition, immortalized cell line from eutopic endometrial stromal cells (hEM15A) cell line derived from patients with EMs was cultured in RPMI-1640 medium enriched with 15% FBS and 1% penicillin–streptomycin (Biological Industries, Israel).

### Plasmids transfection and stable cell line construction

Inoculated eESCs into a 6-well plate at 1 × 10^5^ cells per well. When they reached 70% confluence, transfected them with si-GLS1 (Ribobio, China), si-c-MYC (Ribobio, China), IGF2BP3 overexpression plasmids (Xibei, China), and UCA1 overexpression plasmids (Xibei, China). For transfection, diluted 2.5 μg of DNA and 5 μL Lipo3000 (Invitrogen, USA) respectively in 125 μL Opti-MEM (Gibco, USA). Placed the mixture at room temperature for 20 min. Added it to the 6-well plate. After 8 h, replaced the supernatants and collected the eESCs 48 h post-transfection. The transfection efficiency was shown in Fig. S2. The lentiviruses targeting IGF2BP3 and UCA1 were obtained from Shanghai Gene Chemical Company. Following 72 h of infection, selected the cells using 3.5 µg/ml of puromycin. The desired sequences of shRNA and siRNA were listed in Table S1.

### Immunohistochemical staining

Embedded normal endometrium tisssues (EN) and ectopic endometrial tissues (EC), then slicing and dewaxing them. Performed antigen retrieval using a solution of 3% hydrogen peroxide and citric acid. Incubated the initial antibody (Table S2) at 4 ℃ for 12 h. Applied the secondary antibody (CWBIO, Beijing, China) for 20 min and subsequently treated with DAB. Dehydrated, sealed and dried the slices. Visualized the samples by a microscope (Nikon, Tokyo, Japan) and examined using ImageJ.

### Cell proliferation assay

The CCK8 and EdU assays were employed to evaluate the proliferation of eESCs.

Inoculated the transfected eESCs into a 24-well plate at 2 × 10^4^ cells/mL. Starved the cells in medium without serum for a whole day. Measured by an EdU Assay Kit (Beyotime, C0071S). Calculated the proliferation ability: EdU incorporation rate (%) = EdU positive cells (green)/Hoechst positive cells (blue). Repeated the experiment three times.

Inoculated the transfected eESCs into a 96-well plate at 1 × 10^5^ cells/mL. Categorized the cells into various groups based on different transfection conditions, with 5 parallel wells in each. After cell attached, detected the cell viability using CCK-8 at 0, 24, 48, and 72 h. Added 100 µl of serum-free culture medium containing 10 µl of the CCK-8 solution to each well, and incubated at 37 ℃ for 1–4 h. Measured the OD at 450 nm using an enzyme labeler.

### Cell migration assay

The wound-healing assay and transwell migration assay were used to assess the migratory capability. Prepared 100 μL of serum-free DMEM and inoculated cells at 1 × 10^4^/mL onto the top chamber of an 8 μm pore size insert in the transwell insert. Filled the bottom chamber with 600 μL of DMEM supplemented with 15% FBS. After 24 h, fixed and stained the cells. Used a 200 μL pipette to create scratches. Took images of the scratch areas under a microscope at 0, 24 and 48 h. Wound closure rate (%) = (Scratch distance at 0 h − Scratch distance at 24 h)/Scratch distance at 0 h * 100%.

### Detection of glutamine concentration

Collected the cells into centrifuge tubes and centrifuged. Removed the supernatant. Measured the concentration of Gln within the cells using a reagent kit (ml077293, mlbio, China). The specific content of glutamine can be obtained by measuring the absorption spectrum of each group at 450 nm with a spectrophotometer and then bringing it into the equation of the standard curve. Standard Curve Formula: y = 0.0824x − 0.0012(x represents glutamine content (nmol), and y corresponds to the Δabsorption spectrum).

### RNA isolation and qRT-PCR

Cells or tissues were collected and subjected to total RNA extraction using Trizol (Invitrogen, USA). The reverse transcription of 500 ng of RNA was effectively conducted using a cDNA synthesis kit. Subsequently, 20 ng of cDNA was utilized as a pattern for qRT-PCR with the Top Green qPCR SuperMix kit (TransGen Biotech, China). The primer sequences were sourced from GENEWIZ (GENEWIZ, China) (Table S1).

### RNA stability assay for mRNA lifetime

Inoculated cells into a 12-well plate and allowed them to reach 70–80% confluency after 1 day. Treated the cells with 5 μg/ml actinomycin D (MedChemExpress, HY-17559) to block overall mRNA synthesis. After incubating for 0, 2, 4, and 6 h, extract RNA samples for reverse transcription. Measured the transcription levels of GLS1 and c-MYC mRNA.

### Western blot

Used RIPA lysis buffer and 1% PMSF (Beyotime Biotechnology, China) to lyse cells or tissues. Quantified the samples using the BCA protein assay kit (Beyotime Biotechnology, China). Loaded the samples and then transferred to a PVDF membrane (Millipore, USA). Incubated it with the primary antibody (Table S2) overnight after sealing it for 2 h. Incubated it with the secondary antibody for 2 h. Visualized the bands using an ECL reagent (Epizyme, China).

### Fluorescent in situ hybridization (FISH) and immunofluorescence (IF)

Used the FISH Kit (RiboBio, Guangzhou, China) to perform fluorescence in situ hybridization of RNA. Inoculated cells onto a 24-well chamber slide (Ibidi from Martinsried, Germany). Designed and synthesized specific FISH probes targeting UCA1. Next day, removed DMEM and washed the cells. Fixed the cells with 4% PFA. Blocked the cells with 5% goat serum albumin (Beyotime Biotechnology, China). Treated the cells with Triton X-100 (0.5%) at room temperature for 15 min.Washed the cells. Incubated the slide with denaturing probes or negative controls targeting UCA1, U6, and 18S rRNA at 37 °C overnight. Stained the nucleus with DAPI. Obtained the images by a confocal laser scanning microscope (Examiner. Z1, Carl Zeiss, Germany).

Incubated the slides with IGF2BP3 and c-MYC primary antibodies (Table S2) overnight at 4 ℃. Incubated the slides with fluorescent secondary antibodies at room temperature for 1 h. Obtained the images by a confocal laser scanning microscopy.

### RNA pull-down assay

The RNA pull-down assays were performed by kit (NO. 20164, Thermo Scientifific, Rockford, IL, USA). Biotin-labeled UCA1 and the GLS1 3’UTR sequence were composed by RiboBio (Guangzhou, China) and incubated with cell lysates for 4 h. Next day, the streptavidin magnetic beads were utilized to capture proteins that interacted with the biotin-labeled UCA1 or GLS1 3’UTR. The protein-RNA-bead complexes were gathered and the proteins were extracted. Western blot analysis was performed to examine the obtained proteins (Table S2).

### RNA immunoprecipitation (RIP)

The RIP assay was performed by RIP Kit (#17-700, Millipore, Billerica, MA). Lysed the cells and hatched them at 4 °C overnight with 4 µg of anti-IGF2BP3 antibody (ab179807, Abcam, USA) and c-MYC antibody (ab185656, Abcam, USA). IgG was used as a control. Used qRT-PCR to analyze.

### Animal experiments

Female C57BL/6 mice, 8 weeks old, were obtained from the animal center of our hospital. The model of EMs was induced by intraperitoneal injection of uterine fragments from a single donor mouse into two recipient mice (Gou et al. 2019). A total of 36 recipient mice were established as EMs mouse models. In the experiment group, mice received intraperitoneal injections of 50 μg of BPTES (HY-12683, MCE, China) every 4 days starting from day 3, while the control group received PBS injections. The mice were euthanized on day 5, 9 and 14, and samples were collected. Each group comprised six mice at each time point.

Authorization for the animal experiments was secured from the Ethics Committee (Ethical Number: 2020PS190K). A total of 5 × 10^6^ hEM15A cells were administered subcutaneously into the right armpit of 4–6-week-old female BALB/c nude mice (Changsheng Biosciences, JiLin, China). Tumor growth rate was monitored for four consecutive days. V_tumor_ = π/6 × length × width^^2^. After a period of 3 weeks following injection, the mice were humanely euthanized. Collected the tumor samples.

### Statistical analysis

Each trial was conducted autonomously with 3 replicates. Statistical analysis was carried out using GraphPad Prism 8 (San Diego, USA), and the results were expressed as the average ± SD. For data comparison, the Student’s t-test, one-way ANOVA, and Two-way ANOVA were employed. Statistical significance was defined at *p* < 0.05. Results indicating no significant difference were labeled as "ns" (*p* ≥ 0.05), whereas ****p < 0.0001, ***p < 0.001, **p < 0.01, *p < 0.05 represented significant differences.

## Results

### The expression of IGF2BP3 was elevated in EMs

**Fig. 1:**
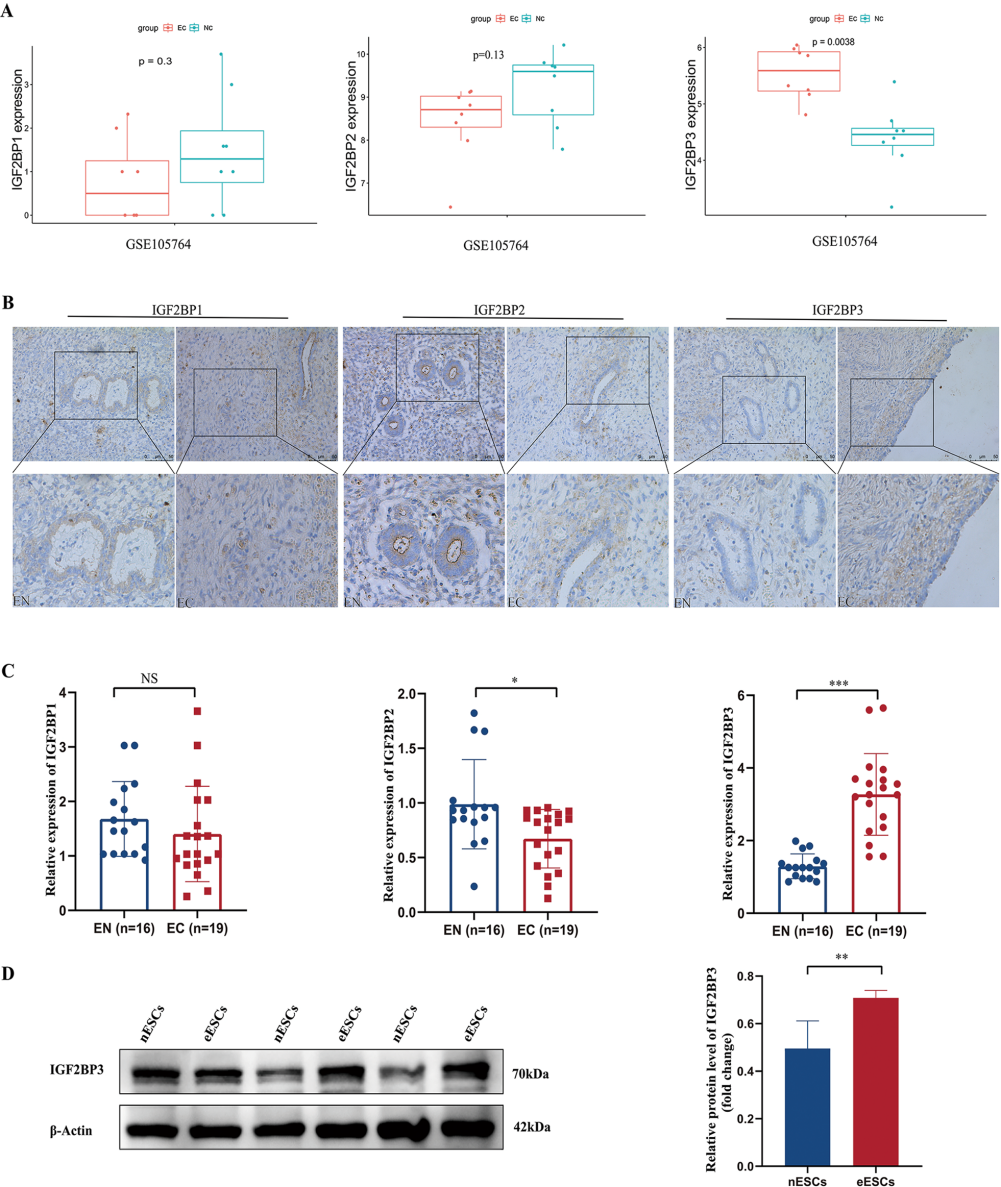
The expression of IGF2BP3 was increased. **A** The mRNA expression levels of IGF2BP1, IGF2BP2 and IGF2BP3 in the GEO database (GSE105764). **B** The protein expression of IGF2BP1, IGF2BP2 and IGF2BP3 in EN and EC. **C** The mRNA levels. **D** IGF2BP3 protein expression in nESCs and eESCs

Previous studies have revealed that IGF2BP3 acted as a possible predictive biomarker for poor outcomes in CCC (Bi et al. 2016). Given the potential progression of EMs to CCC and the shared molecular underpinnings of these two conditions, we embarked on an investigation into the involvement of IGF2BPs in EMs progression. To delve into the function of the IGF2BP family in EMs, we utilized the GEO dataset (GSE105764) (https://www.ncbi.nlm.nih.gov/geo/) to analyze the differential expression of IGF2BPs between EC and EN. The lncRNA and mRNA expression profiles (GSE105764) related to ectopic and eutopic endometria of ovarian endometriosis were selected, The Limma package (version:3.40.2, http://www.bioconductor.orgackages/release/bioc/html/limma.html) in R software was used to analyze the differential expression of mRNA. The findings revealed a notable increase in the mRNA levels of IGF2BP3 in EC compared to EN. Conversely, no statistically great distinctions were observed in the manifestation of other members of the IGF2BPs (Fig. 1A). To validate the results obtained from our bioinformatics analysis, we performed IHC staining. The staining intensity (IOD values) showed a significant increase in the protein abundance of IGF2BP3 in EC compared to EN (Fig. 1B). We conducted qRT-PCR analysis to further validate our findings. The results demonstrated a higher expression of IGF2BP3 in EC (n = 19) compared to EN (n = 16). Conversely, IGF2BP2 demonstrated a slight decrease, while IGF2BP1 showed no dramatic difference (Fig. 1C). The western blot analysis revealed a significantly higher expression level of IGF2BP3 in eESCs compared to nESCs (Fig S1A and Fig. 1D).

### IGF2BP3 promotes the proliferation and migration of eESCs

**Fig. 2:**
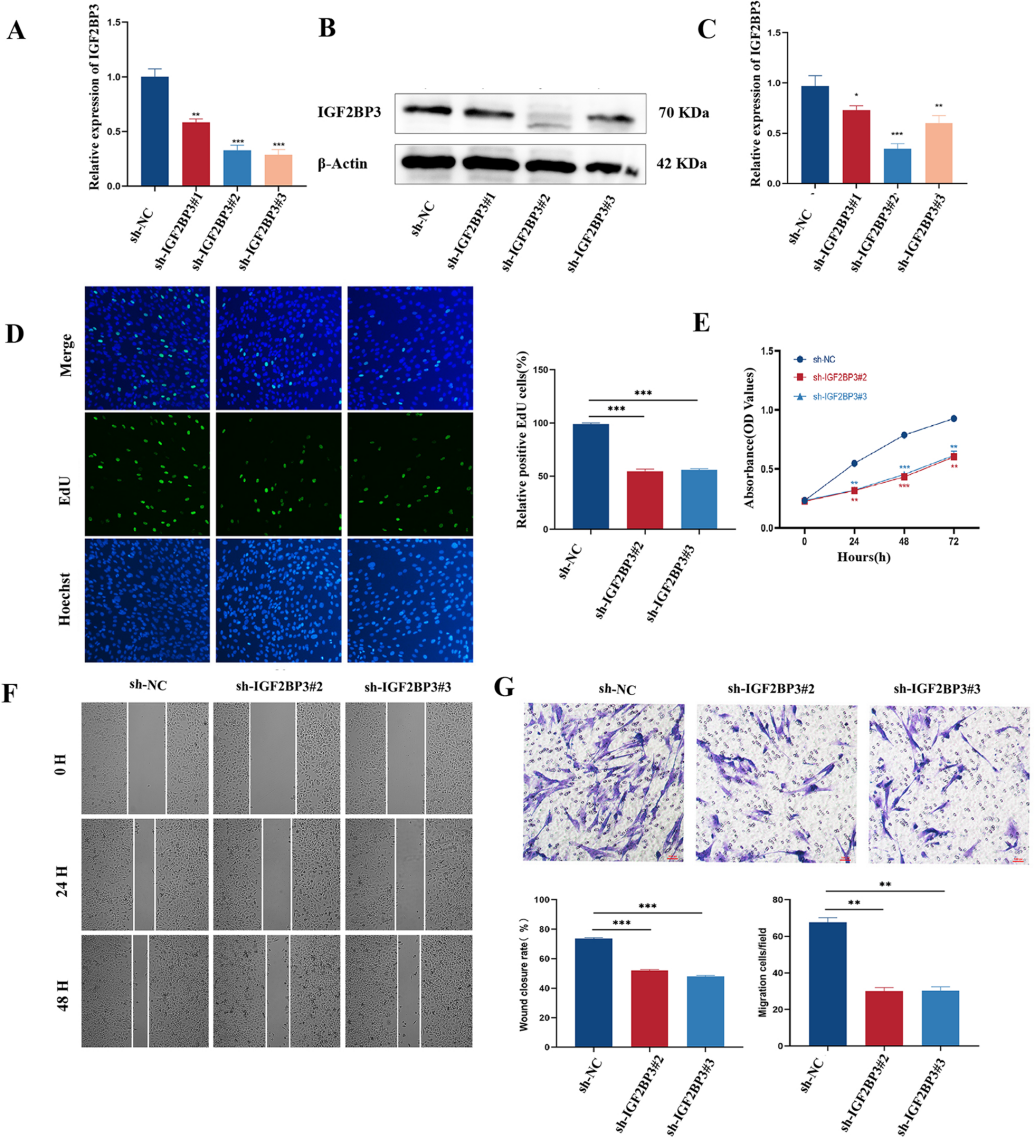
IGF2BP3 promoted the proliferation and migration of eESCs. **A–C** The knockdown efficiency of IGF2BP3. **D**, **E** The proliferation capacity after transfection of eESCs. **F**, **G** The migration ability after transfection of eESCs

To delve into the involvement of IGF2BP3 in the advancement of EMs, we employed lentivirus to effectively inhibit the expression of IGF2BP3 in eESCs. Following validation of efficiency (Fig. 2A–C), we assessed the impact of IGF2BP3 knockdown on the biological characteristics by the EdU assays (Fig. 2D–E), the CCK8 (Fig. 2F), the wound healing assays (Fig. 2G–H) and the transwell migration assays (Fig. 2I–J). Our results revealed that the stable reduction of IGF2BP3 notably impeded the proliferation and migration capacities. Subsequently, we conducted the overexpression of IGF2BP3 in eESCs and confirmed its effectiveness (Fig. S2A). Overall, these findings indicated an essential function for IGF2BP3 in modulating the proliferation and migration of eESCs.

### UCA1 binds to IGF2BP3 in the cytoplasm and affects proliferation and migration of eESCs

**Fig. 3:**
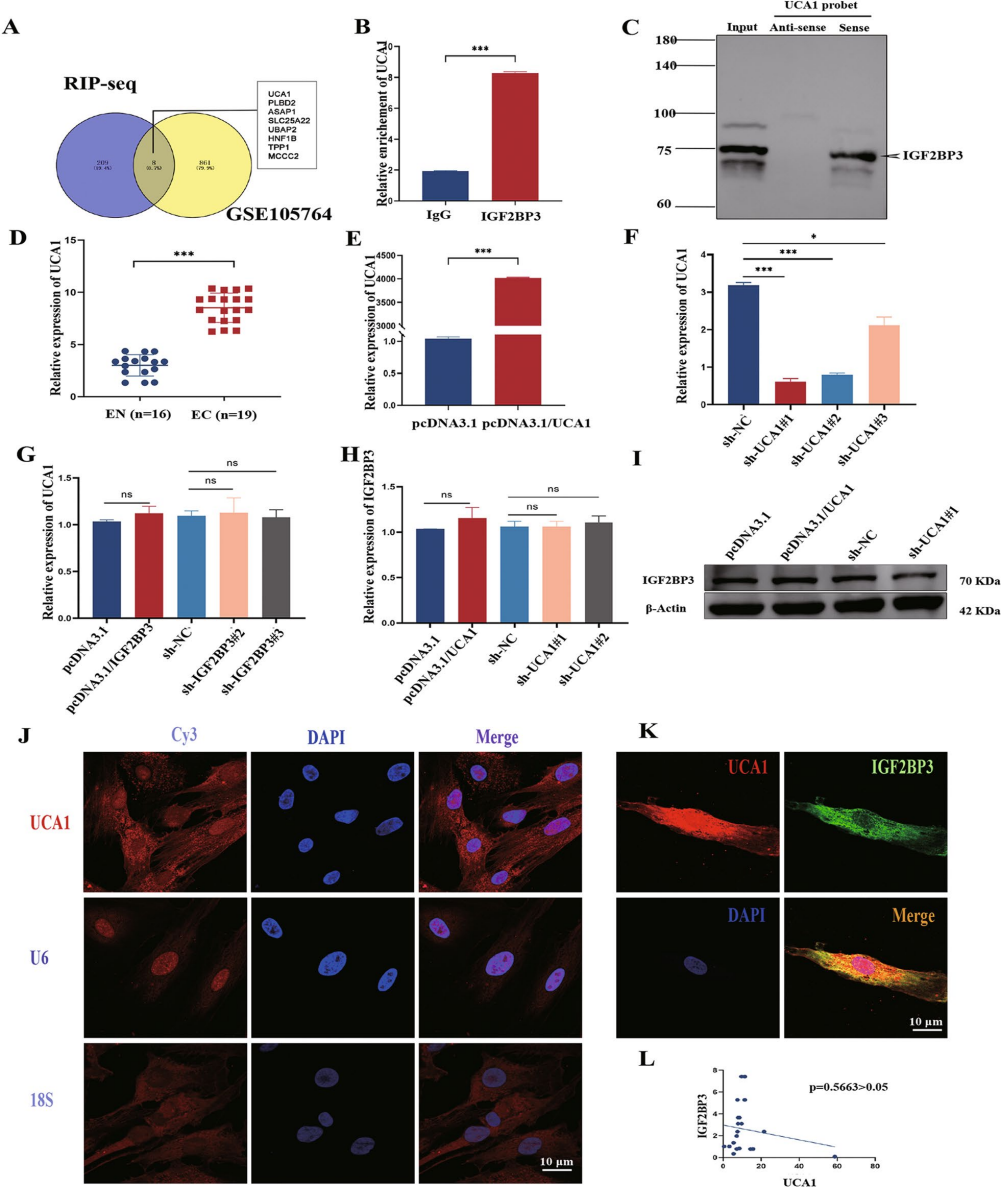
UCA1 bond to IGF2BP3 in the cytoplasm and affected proliferation and migration of eESCs. **A** lncRNAs that was bind to IGF2BP3. **B** RIP-qPCR assays to confirm the interaction between IGF2BP3 and UCA1. **C** Western blot assays to confirm the interaction. **D** qRT-PCR confirmed the levels of UCA1 in EN and EC. **E** The levels of UCA1 were examined following transfection with a pcDNA3.1 overexpression plasmid in eESCs. **F** UCA1 expression levels were knocked down using 3 shRNAs in eESCs. **G** qRT-PCR assays revealed that there was no significant regulation of UCA1 in either IGF2BP3-overexpressing or IGF2BP3-silenced eESCs. **H**, **I** The mRNA and protein expression levels of IGF2BP3. **J** Representative images of UCA1 localization in eESCs. **K** IF-FISH staining experiments indicated that UCA1 (red) located together with IGF2BP3 (green) in the cytoplasm of eESCs. **L** The correlation between UCA1 and IGF2BP3 was analyzed by detecting the expression of mRNA in EC

IGF2BP3 primarily acts as an RNA-binding protein. We utilized the GEO database (GSE47597) to screen and identify 209 potential mRNAs that interacted with IGF2BP3. This dataset was obtained by performing RIP-seq of IGF2BP3 using an anti-IGF2BP3 antibody or normal rabbit IgG in extracts from the fibronectin-stimulated pancreatic cancer cell line. Subsequently, we identified 861 differentially expressed lncRNAs in EC and EN (GSE105764). The intersection analysis revealed that among the differentially expressed lncRNAs, eight showed potential binding abilities to IGF2BP3 in eESCs (Fig. 3A). RIP experiments confirmed an interaction between IGF2BP3 and UCA1, indicating a specific binding between UCA1 and IGF2BP3 protein (Fig. 3B). Furthermore, the RNA pull-down assay demonstrated that the UCA1 sense RNA probe exhibited a higher capacity to pull down IGF2BP3 compared to the anti-sense RNA probe (Fig. 3C). The qRT-PCR results also provided additional evidence supporting the substantial increase in UCA1 expression in EMs (Fig. 3D). The efficiency of transfection of overexpressing UCA1 plasmid and sh-UCA1 lentivirus into eESCs were evaluated (Fig. 3E, F). To further investigate the relationship between UCA1 and IGF2BP3, qRT-PCR analysis was performed. Remarkably, the results clearly indicated that the manipulation of IGF2BP3 did not significantly impact the expression of UCA1 (Fig. 3G). Additionally, UCA1 knockdown or overexpression had little impact on the expression of IGF2BP3 (Fig. 3H, I). Therefore, while UCA1 did bind to IGF2BP3, it had limited influence on the regulation of mRNA or protein expression levels. Furthermore, FISH-IF assay showed that UCA1 existed in both the nucleus and the cytoplasm (Fig. 3I). Meanwhile, the interaction between IGF2BP3 and UCA1 predominantly occurred in the cytoplasm (Fig. 3J). Moreover, there was no notable association observed between the expression levels of IGF2BP3 and UCA1 in 19 EC patients (Fig. 3K).

**Fig. 4:**
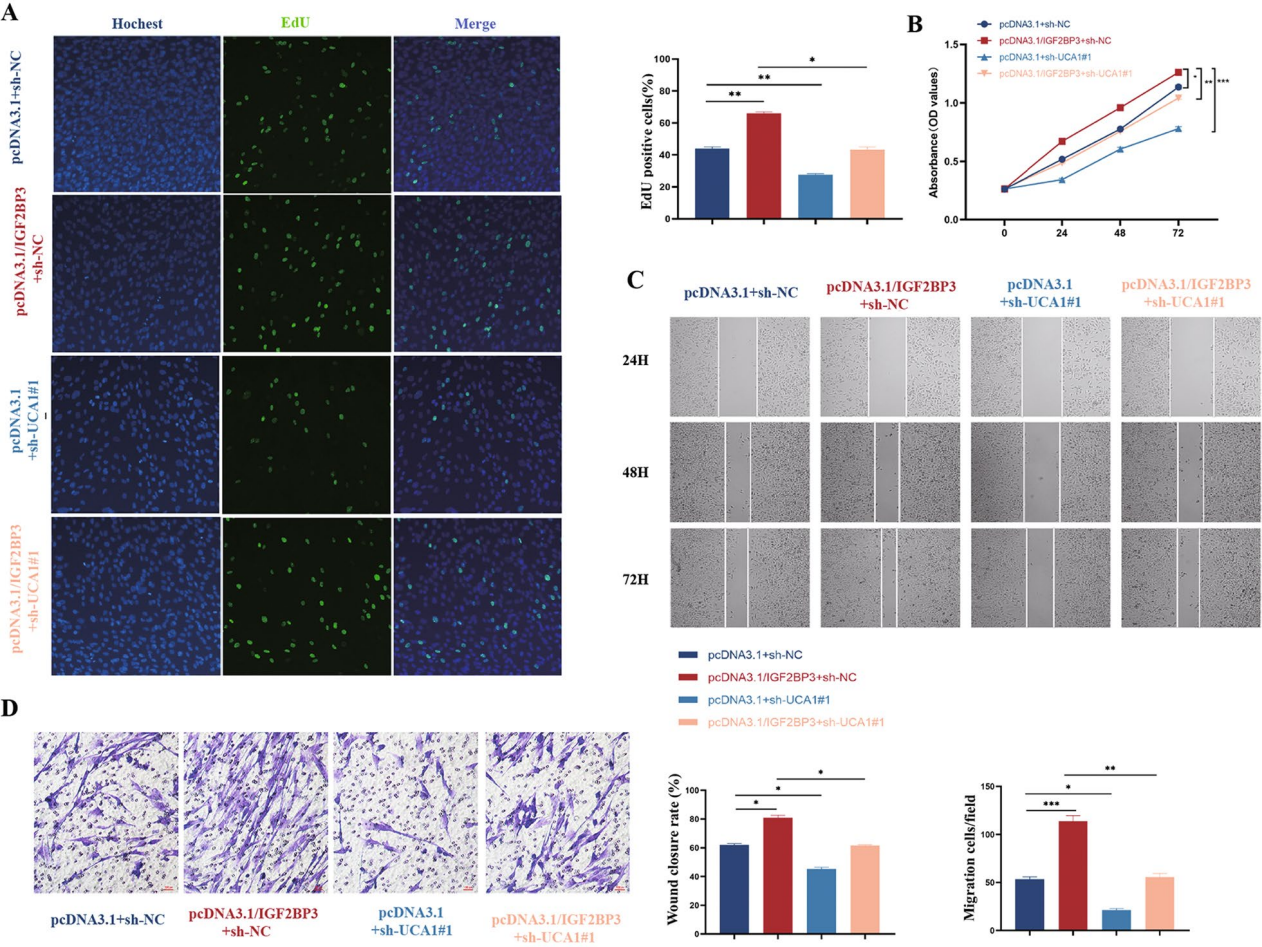
IGF2BP3 exerted its pro-proliferative effect by interacting with UCA1 of eESCs in vitro. **A**, **B** Edu and CCK-8 assays indicated the proliferation ability of eESCs after transfection with pcDNA3.1 + sh-NC, pcDNA3.1/IGF2BP3 + sh-NC, pcDNA3.1 + sh-UCA1#1 and pcDNA3.1/IGF2BP3 + sh-UCA1#1 as indicated. **C**, **D** Wound healing assay and transwell migration assay show migration ability after transfection with pcDNA3.1 + sh-NC, pcDNA3.1/IGF2BP3 + sh-NC, pcDNA3.1 + sh-UCA1#1 and pcDNA3.1/IGF2BP3 + sh-UCA1#1 as indicated

To investigate the potential cooperative regulation of biological functions in eESCs by UCA1 and IGF2BP3, we introduced an IGF2BP3 overexpression plasmid (pcDNA3.1/IGF2BP3) and its negative control (pcDNA3.1) into eESCs that were stably transfected with sh-UCA1 lentivirus. The results demonstrated that the overexpression of IGF2BP3 successfully reversed the suppressive impacts of UCA1 on the proliferation (Fig. 4A, B) and migration (Fig. 4C, D) abilities of eESCs.

### IGF2BP3 upregulates GLS1 expression in eESCs by interacting with UCA1

**Fig. 5:**
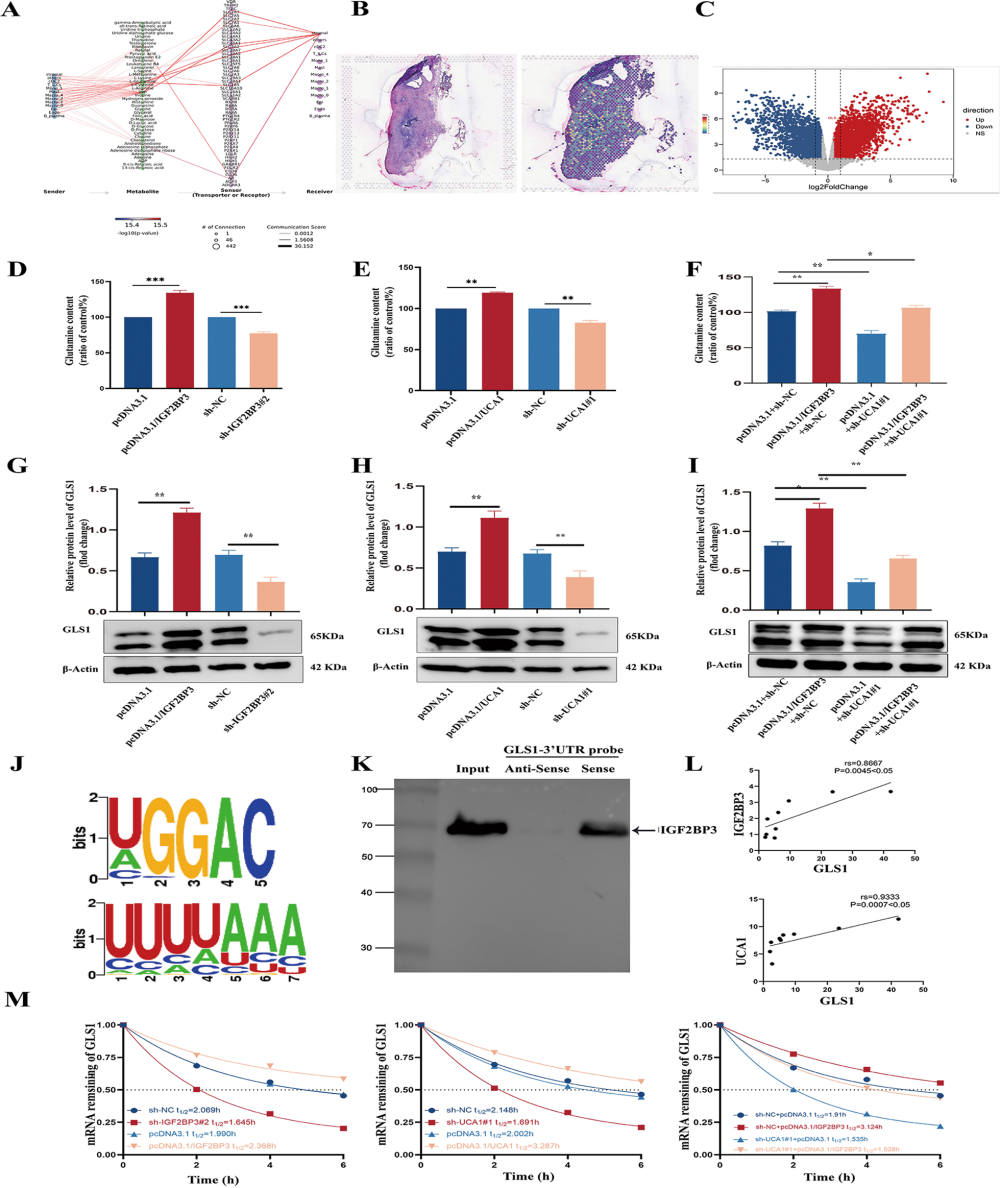
IGF2BP3 up-regulated GLS1 expression in eESCs by interacting with UCA1. **A** The GEO dataset (GSE179640) using the mebocost algorithm in EC revealed that stromal cells uptake glutamine from macrophages to sustain cellular metabolic activity. **B** Single-cell spatial omics form the GEO dataset (GSE213216) through spatial transcriptomic analysis demonstrated the expression distribution of GLS1 mRNA in EC. **C** The volcano plot using another GEO dataset (GSE105764) showed the expression of GLS1 between EN and EC. **D**, **E** The spectrophotometer was used to determine the glutamine uptake content in eESCs that were transfected with IGF2BP3-overexpressing plasmid and IGF2BP3-silenced lentivirus (**D**), as well as UCA1-overexpressing plasmid and UCA1-silenced lentivirus (**E**). **F** The spectrophotometer was used to measure the Glutamine uptake content in eESCs that were transfected with a lentivirus containing a non-targeting control sequence or UCA1-silenced lentivirus and co-transfected with IGF2BP3-overexpressing plasmid or matching control plasmid. **G**, **H** Western blot assays showed the expression of GLS1 in eESCs transfected with IGF2BP3-overexpressing plasmid and IGF2BP3-silenced lentivirus (**G**) as well as UCA1-overexpressing plasmid and UCA1-silenced lentivirus (**H**). **I** Western blot assays were executed to measure the levels of GLS1 expression in eESCs of different transfection ways as before. **J** Predicted binding motifs for IGF2BP3. **K** Verified the association between IGF2BP3 protein and the GLS1 3' UTR regions by RNA pull assays and western blot. **L** Correlation analysis was conducted to examine the mRNA expression of IGF2BP3 and GLS1, as well as UCA1 and GLS1, in EC. **M** qRT-PCR was utilized to detect the half-life of GLS1 in eESCs subjected to various treatments involving IGF2BP3 and UCA1. These treatments included transfection of an IGF2BP3-overexpressing plasmid and an IGF2BP3-silenced lentivirus, as well as transfection of a UCA1-overexpressing plasmid and a UCA1-silenced lentivirus. Additionally, a negative control lentivirus or UCA1-silenced lentivirus was co-transfected with an IGF2BP3-overexpressing plasmid or the corresponding control plasmid

By using single-cell RNA sequencing data from the GEO dataset (GSE179640) and applying the MEBOCOST algorithm, we quantitatively inferred the metabolic product sensor communication pathways within stromal cells. The processing of the single-cell sequencing dataset applied the standard workflow of the Seurat package (https://github.com/satijalab/seurat), cell typing was based on consensus markers, and potential metabolic interactions were quantified using MEBOCOST (https://github.com/zhengrongbin/MEBOCOST). The findings revealed that glutamine, which maintaind cell life activities in stromal cells, primarily originated from macrophages (Fig. 5A). Furthermore, by conducting spatial transcriptome analysis of the GEO dataset (GSE213216), we observed the expression of the critical enzyme GLS1 involved in glutamine metabolism within endometrial lesions (Fig. 5B), and the data was visualized using the Seurat package. We also investigated the expression of GLS1 in EC and EN using another GEO dataset (GSE105764) (Fig. 5C). Additionally, insights from the GSE231938 dataset revealed a positive association between GLS1 and IGF2BP3 in non-small cell lung cancer, prompting further exploration into the interplay among GLS1, IGF2BP3 and UCA1. After altering the expression levels of UCA1 and IGF2BP3, we observed changes in both glutamine consumption and GLS1 expression levels (Fig. 5D–F). In order to delve deeper into the underlying mechanism of the UCA1 and IGF2BP3 interaction in the regulation of GLS1 expression, a rescue experiment was conducted. The results demonstrated that down-regulation of UCA1 effectively prevented the increase in GLS1 expression induced by IGF2BP3 in eESCs (Fig. 5G–I). Based on previous studies, it has been suggested that the IGF2BP3 protein selectively bind to the conserved sequences "UGGAC" or "UUUUAAA" present in the 3'UTR. To investigate the potential protein-RNA interactions between IGF2BP3 and GLS1, we performed computational analysis using the starBase website (Fig. 5J). For the RNA pull-down experiment, a 3'UTR fragment labeled with biotin of GLS1 containing the predicted binding pattern was used. The outcomes revealed a notable increase in the binding of the IGF2BP3 protein to the GLS1 3'UTR sequence as compared to the negative control (Fig. 5K). Our study discovered a positive association among the expression levels of GLS1, IGF2BP3 and UCA1 in 9 patients with EMs (Fig. 5L). To further investigate the impact of the UCA1/IGF2BP3 interplay on the stability of GLS1 mRNA, we conducted RNA stability assays. The alterations in both UCA1 and IGF2BP3 affected the stability of GLS1 mRNA. Additionally, rescue experiments indicated that reducing the expression of UCA1 counteracted the increased stability of GLS1 mRNA observed in eESCs overexpressing IGF2BP3 (Fig. 5M). Overall, these findings provided compelling evidence that underscored the crucial function of UCA1 in the IGF2BP3-mediated prolongation of GLS1 mRNA half-life.

### IGF2BP3 exerts its pro-proliferative and pro-migration effect in eESCs through GLS1

**Fig. 6:**
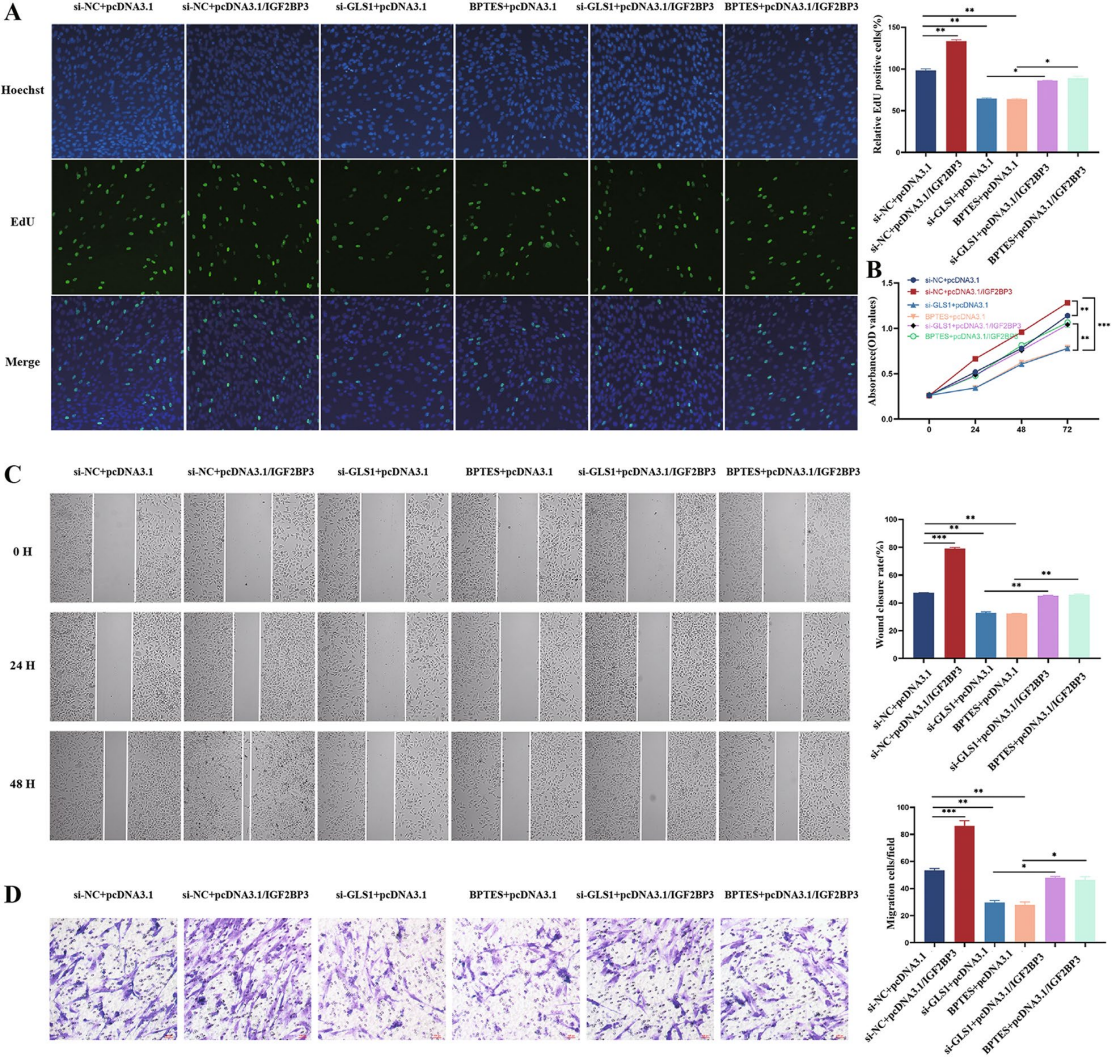
IGF2BP3 exerted its pro-proliferative effect in eESCs via GLS1. **A**, **B** The proliferation of eESCs overexpressing IGF2BP3 combined with si-GLS1 or BPTES was assessed through Edu assays and CCK-8 assays. **C**, **D** Wound healing assays and transwell migration assays revealed that eESCs with IGF2BP3-overexpression exhibited increased migration compared to cells with GLS1-silenced or demonstrate with BPTES

GLS1 plays a pivotal role in various cancers, particularly in ovarian cancer and endometrial cancer (Wu et al. 2021). Subsequently, siRNAs were transfected into eESCs to assess their efficiency (Fig S2B). The inhibitor BPTES was employed to inhibit GLS1 function by reducing its enzyme activity. Results from the rescue experiments indicated that both knockdown of GLS1 and treatment with BPTES resulted in inhibited proliferation (Fig. 6A, B) and migration (Fig. 6C, D) of eESCs. Importantly, these effects were mediated by IGF2BP3, suggesting that IGF2BP3 promoted eESC progression by regulating GLS1 function.

### UCA1 promotes GLS1 expression by stabilizing c-MYC levels

**Fig. 7:**
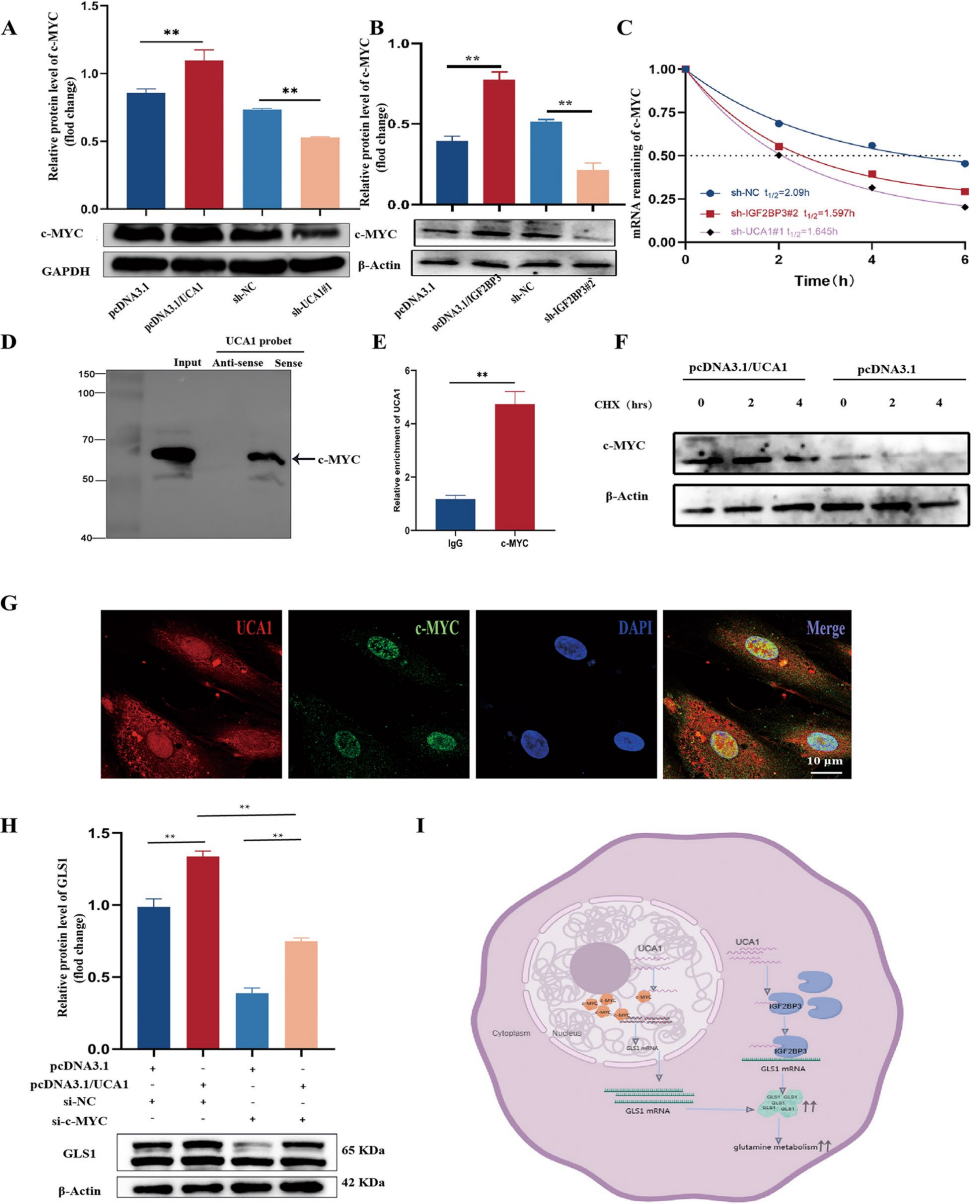
UCA1 promoted GLS1 expression by stabilizing c-MYC levels. **A**, **B** Quantified the protein abundance of c-MYC in eESCs transfected with UCA1-overexpressing plasmid and UCA1-silenced lentivirus (**A**), and IGF2BP3-overexpressing plasmid and IGF2BP3-silenced lentivirus (**B**). **C** Used qRT-PCR to evaluate the expression of c-MYC mRNA in eESCs transfected with UCA1-silenced lentivirus and IGF2BP3-silenced lentivirus, subsequently managed with actinomycin D (5 μg/ml) for the specified durations. **D** The relationship between c-MYC protein and UCA1. **E** RIP assay. **F** Used western blot to explore the influence of CHX treatment on UCA1-overexpressing plasmid mediated changes in c-MYC protein levels. **G** RNA-FISH combined with Immunofluorescence staining experiments showed that UCA1 (red) located together with c-MYC (green) in the nucleus of eESCs. **H** Western blot was performed to measure the expression of GLS1 in eESCs. **I** A graphical representation was created to depict the regulation of the IGF2BP3/UCA1/c-MYC/GLS1 axis in EM

Numerous researches have shown that UCA1 was crucial in c-MYC. The western blot outcomes revealed that both UCA1 and IGF2BP3 positively regulated the expression of c-MYC (Fig. 7A, B). Further investigation using actinomycin D delved into the involvement of UCA1 and IGF2BP3 in maintaining c-MYC stability. The results indicated that knockdown of IGF2BP3 and UCA1 led to a reduction in c-MYC mRNA stability (Fig. 7C). Moreover, UCA1 was shown to interact with c-MYC protein through RNA pull-down and RIP assays (Fig. 7D, E). Based on the data presented, it was assumed that UCA1 has the capability to bind to c-MYC levels post-transcriptionally. It was speculated that this binding could offer protection against the degradation of c-MYC. When eESCs were exposed to the protein synthesis inhibitor CHX, overexpression of UCA1 was observed to prevent the breakdown of c-MYC protein (Fig. 7F). Furthermore, the connection of UCA1 and c-MYC was confirmed in the nucleus via a FISH-IF assay (Fig. 7G). Previous studies have presented compelling evidence demonstrating that c-MYC had the ability to bind to the specific region of GLS1 promoter, leading to a significant augmentation in its expression. In this context, the upregulation of UCA1 resulted in elevated mRNA expression of GLS1, a response was found to be reversible upon the inhibition of c-MYC (Fig. 7H). Collectively, these findings indicated that UCA1 facilitated glutamine metabolism in eESCs through the IGF2BP3/c-MYC/GLS1 signaling pathway (Fig. 7I).

### Blocking the UCA1/IGF2BP3/GLS1 axis inhibited the progression of EMs in vivo

**Fig. 8:**
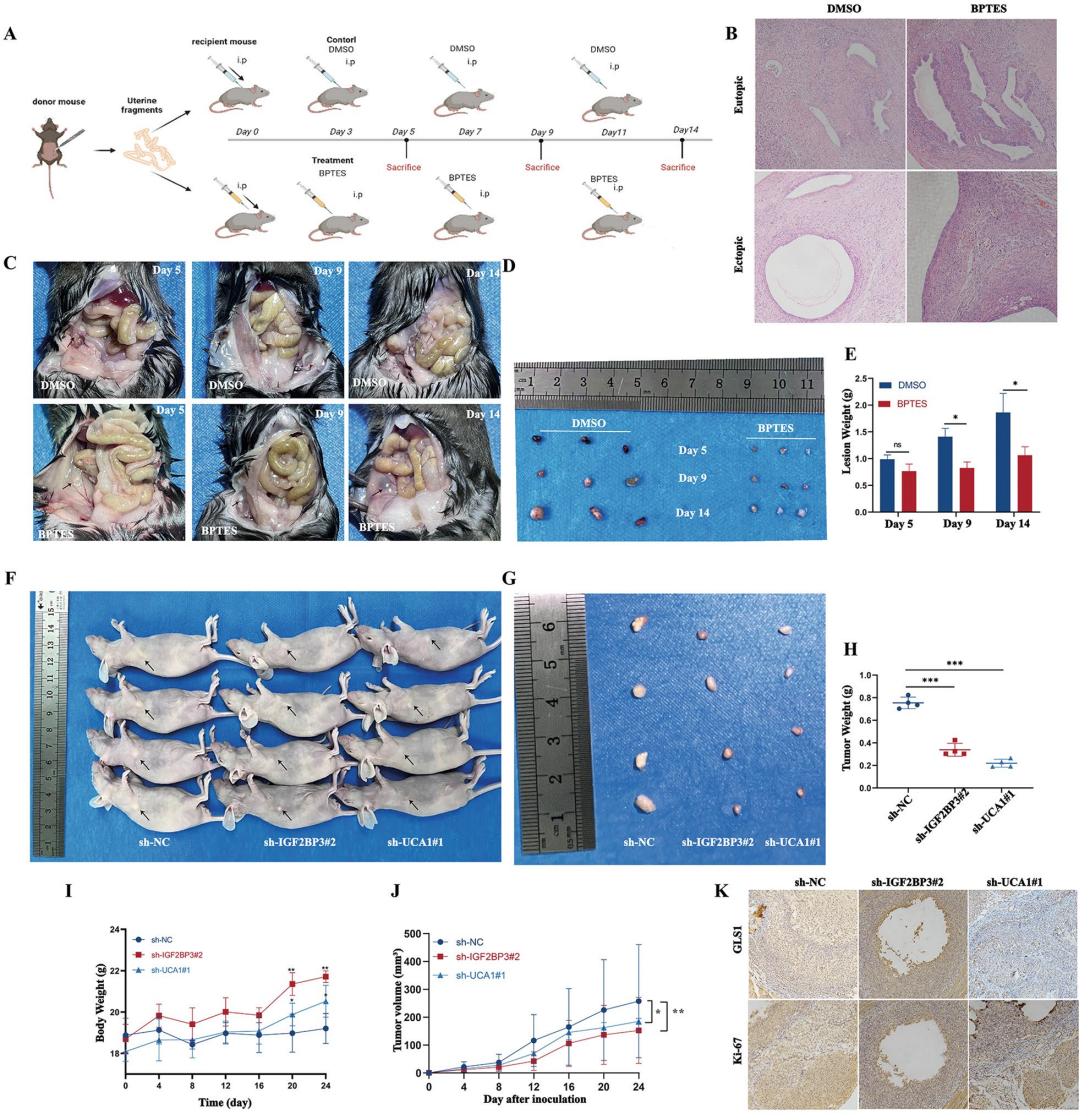
Blocking the UCA1/IGF2BP3/GLS1 axis inhibited the progression of EM in vivo. **A** The circuit diagram for the creation of the mouse EM model. Created by BioRender.com. **B** HE staining of GLS1 in uterine tissues and ectopic lesions in the models following treatment with either DMSO or BPTES. Scale bar: 100 µm. **C**, **D** Visible lesions of the model of EM after treatment. **E** The weight of ectopic lesions. (F-I) Representative images of subcutaneous tumors from mice (**F**, **G**) injected with control lentivirus cells (left), IGF2BP3-silenced lentivirus cells (middle) or UCA1-silenced lentivirus cells (right). The mean weight of tumors was measured (**H**); body weight growth curves (**I**) and tumor volume growth curves (**J**) were plotted every 4 days until 24 days. **K** The levels of GLS1 protein and Ki-67 index of different groups by IHC. Scale bar: 50 μm

To assess the influence of GLS1 on the progression of EMs in vivo, EMs mice models were established. All of the mice were separated into two categories: the control group and the BPTES group (Fig. 8A). HE staining of uterus and ectopic lesions demonstrated a significant decrease in disease severity in the BPTES group (Fig. 8B–D). When compared to the control group, the BPTES group showed reductions in both lesion weight and size (Fig. 8E). To validate the roles of IGF2BP3 and UCA1 in EMs in vivo, subcutaneous xenograft nude mice models were established. However, due to the limited proliferative capacity of eESCs, an immortalized cell line (hEM15A) was used instead. In line with their in vitro findings, down-regulation of IGF2BP3 and UCA1 in hEM15A cells resulted in a decrease in subcutaneous xenograft growth, weight and volume, as well as a corresponding reduction in the weight of the nude mice.

Immunohistochemistry experiments revealed that the down-regulation of IGF2BP3 and UCA1 led to decreased expression levels of GLS1 and Ki67 (Fig. 8K). In conclusion, these in vivo studies validated that IGF2BP3 and UCA1 promoted the proliferation of hEM15A cells. These findings provided a basis for further exploration of a novel combination therapy for EMs.

## Discussion

EMs is a long-term inflammatory gynecologic disease characterized by the presence of ectopic endometrial tissue. It has been believed to primarily affect the pelvic, but recent studies have found that it can affect liver and adipose tissue metabolism, resulting in widespread inflammation throughout the body. Changes in gene expression within the brain have also been observed, which contribute to heightened pain sensitivity and the development of emotional disorders (Taylor et al. 2021). Although the exact cause of endometriosis is still unclear, researchers have been investigating the role of RBPs in its development. RBPs have the ability to recognize multiple RNA transcripts and form regulatory networks that are crucial for maintaining cellular balance. Dysfunction of RBPs has been implicated in development of various diseases. One particular RBP, IGF2BP3, has been linked with metabolic reprogramming in various diseases like gastric cancer, lung cancer, and glioma (Lin et al. 2023; Wang et al. 2023; Fang et al. 2023). Our observations have shown that the metabolism of glutamine in endometriotic cells undergoes reprogramming, resulting in heightened activity of important metabolic enzymes. Previous studies have confirmed the involvement of different transcription factors (TFs) and signaling pathways in the reprogramming of glutamine metabolism in other diseases (Najumudeen et al. 2021). However, the extent of their contribution to endometriosis remains uncertain. Based on our research, we have discovered that the IGF2BP3/UCA1/c-MYC/GLS1 axis was crucial to stimulate the proliferation and migration of endometriotic cells through its regulation of glutamine metabolism. IGF2BP3 is an RNA-binding protein that plays a vital role in regulating the stability of mRNAs. Increased expression of IGF2BP3 contributed to the development of tubular cell fibrosis by directly binding to β-catenin mRNA, enhancing its stability and preventing degradation (Song et al. 2023). In prostate cancer, a circular RNA called hsa_cir_0003258 bound to IGF2BP3 in the cytoplasm, leading to increased stability of HDAC4 mRNA and promoting cancer metastasis (Yu et al. 2022). Recent studies have also highlighted the involvement of lncRNAs in interacting with IGF2BP3, influencing the progression of cancer (Hanniford et al. 2020; Xia et al. 2022). In male non-small cell lung cancer cells, linc-SPRY3-2/3/4 has been found to interact with IGF2BP3, resulting in a decrease in the stability of specific IGF2BP3 binding mRNAs like anti-apoptotic HMGA2 mRNA and oncogenic c-MYC mRNA (Brownmiller et al. 2020). This study examined the expression of IGF2BP1/2/3 of the IGF2BP family at the tissue level and observed an upregulation of IGF2BP3 in EMs. This upregulation was linked to the promotion of the progression of eESCs, indicating that IGF2BP3 may have diagnostic value for EMs cancer.

Through analyzing the RIP-seq dataset and GEO database,we identified UCA1 as an interaction partner of IGF2BP3, which was initially recognized as a promoter of bladder cancer (Li et al. 2015). Liu et al. have confirmed that UCA1 promoted the proliferation of eESCs, while inhibiting autophagy and apoptosis (Jiang et al. 2020). Subcellular localization is a crucial factor in determining the biological function of lncRNAs. The FISH experiments indicated its presence in both the cytoplasm and the nucleus, although its effects were predominantly exerted in the cytoplasm. Our research outcomes revealed that knockdown of UCA1 counteracted the actions of IGF2BP3 in the proliferation and migration of eESCs. This was achieved through the binding of UCA1 to IGF2BP3 in the cytoplasm, thereby disrupting the mRNA stability of downstream genes. Importantly, this process did not affect the expression level of IGF2BP3.

To delve deeper into the downstream genes influenced by the UCA1/IGF2BP3 axis in EMs, we found obvious communication pathway involving glutamine metabolism between eESCs and macrophages by using single-cell RNA-seq data from the GEO dataset (GSE179640) in EMs. Recent literature indicated that both IGF2BP3 and UCA1 were closely associated with glutamine reprogramming (Fang et al. 2023; Zhao et al. 2022a,b). Further investigation demonstrated that UCA1 and IGF2BP3 together enhanced the stability of GLS1 mRNA. Additionally, IGF2BP3 played a crucial role in regulating post-transcriptional gene expression (Tang et al. 2023; Yang et al. 2023a, b). Starbase revealed that there was a direct binding interaction between IGF2BP3 and the 3'-UTR region of the GLS1 gene. This binding interaction allowed IGF2BP3 to exert an independent influence on the stability of GLS1 mRNA by interacting with its 3'-UTR region.

Recent studies have shown a significant increase in β-hydroxybutyric acid and glutamine levels, alongside a decrease in tryptophan levels, in the serum samples of women with EMs. Furthermore, these studies have observed an alteration in pathways related to nitrogen metabolism (Murgia et al. 2021). Glutamine, which falls under the classification of nonessential/conditionally indispensable amino acid, is involved in essential and diverse biological processes. It serves as a crucial source of nitrogen for the synthesis of amino acids and nucleotides. Furthermore, glutamine acts as a carbon provider, helping to renew the tricarboxylic acid cycle (TCA cycle) and playing a part in the biosynthesis of lipids (Altman et al. 2016; Jin et al. 2023). Glutamine metabolism and its associated metabolic pathways, such as those involving glutamine carriers, glutaminase, amino acid transferase, and oxidative stress balance, play a vital role in supporting the survival of cancer cells (Jin et al. 2023; Matés et al. 2020). Previous research has established the crucial role of GLS1 in the metabolic behaviors of cancer cells, facilitating fast growth, cell viability, and immune escape (Chen et al. 2023). However, the presence and influence of GLS1 in eESCs remain unexplored. In this research, we discovered that IGF2BP3 could enhance the proliferation and glutamine metabolism of GLS1-mediated cell in eESCs through its interaction with UCA1. Moreover, preceding studies have indicated that c-MYC interacts with the promoter sequences of GLS1 to iniciate transcriptional activition (Liu et al. 2019; Zeng et al. 2023). In accordance with the evidence concerning GLS1, we noted that the mRNA expression of c-MYC can be elevated by UCA1 and IGF2BP3. Remarkably, this investigation revealed a direct interaction between UCA1 and c-MYC protein, leading to its stability. This interaction consequently resulted in enhanced GLS1 transcription and subsequent activation of glutamine metabolism. As a whole, these collective outcomes revealed that UCA1 drived the promotion of glutamine metabolism mediated by GLS1 in eESCs through its interaction with IGF2BP3 and c-MYC. Nevertheless, the mechanism by which UCA1 binds to IGF2BP3 and influences the stability of downstream target mRNA is an aspect that we will thoroughly investigate in the future studies.

## Conclusion

All in all, it was observed that the expression of IGF2BP3 was detected in EMs. It was found to enhance the proliferation and migration of EMs. In terms of mechanism, we firstly revealed that IGF2BP3 bond directly to UCA1, thereby contributing to the stability of GLS1 mRNA levels.

Furthermore, it was discovered that UCA1 enhanced the function of IGF2BP3 through direct binding, without influencing its expression. Additionally, UCA1 was observed to interact with and maintain mRNA and protein levels of c-MYC. Moreover, it was also demonstrated to activate the expression of GLS1 by facilitating its transcription. This study offered a novel perspective on the underlying mechanisms of EMs and presented new possibilities for developing innovative treatment strategies.

### Supplementary Information

Supplementary Material 1.

Supplementary Material 2.

Supplementary Material 3.

Supplementary Material 4.

## Data Availability

Data partaining to this paper can be obtained by contacting the corresponding author.

## Abbreviations

BSA: Bovine serum albumin
CCC: Ovarian clear cell carcinoma
DAB: Diaminobenzidine
EC: Ectopic endometrial tissues
eESCs: Ectopic endometrial stromal cells
Ems: Endometriosis
EN: Normal endometrium tisssues
FISH: Fluorescence in situ hybridization
GLS1: Glutaminase 1
HE: Hematoxylin-eosin staining
hEM15A: Immortalized cell line from eutopic endometrial stromal cells
IF: Immunofuorescence
IGF2BP1/2/3: Insulin-like growth factor 2 mRNA-binding proteins 1/2/3
IHC: Immunohistochemical
IOD: Mean integrated optical density
lncRNAs: Long non-coding RNAs
nESCs: Normal endometrial stromal cells
qRT-PCR: Quantitative real time PCR
RBPs: RNA-binding proteins
RIP: RNA immunoprecipitation
siRNAs: Small interfering RNAs
TCA: Tricarboxylic acid cyc
TFs: Transcription factors
TME: Tumor microenvironment
UCA1: Urothelial carcinoma-associated 1
