# Supplementary figures and images for: IGF2BP3 promotes glutamine metabolism of endometriosis by interacting with UCA1 to enhances the mRNA stability of GLS1
Source: Mol Med. 2024 May 17;30:64. doi: 10.1186/s10020-024-00834-7 (PMC11102260; doi:10.1186/s10020-024-00834-7)

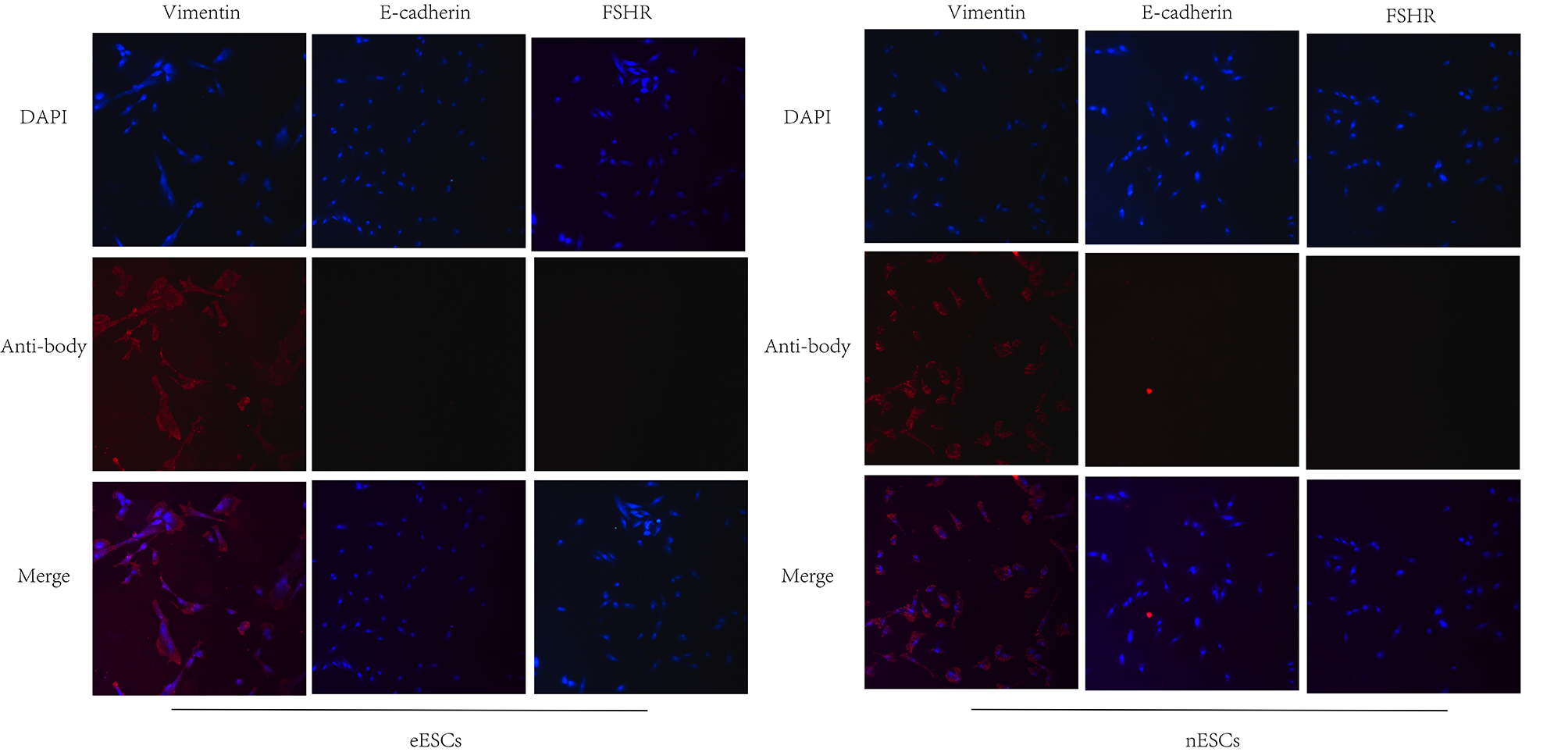

Supplement: Supplementary file 1 — Supplementary Material 1. [file 10020_2024_834_MOESM1_ESM.tif]

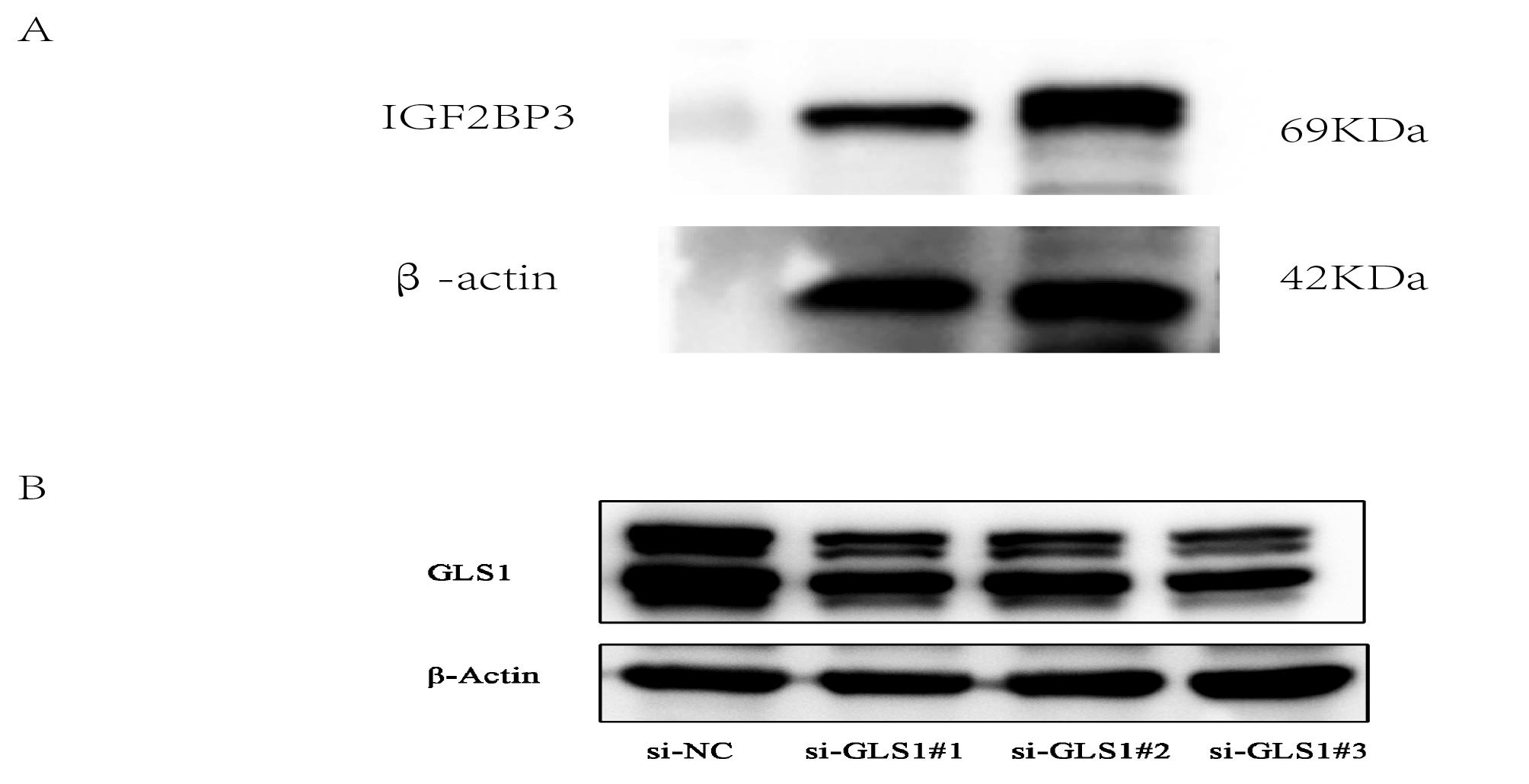

Supplement: Supplementary file 2 — Supplementary Material 2. [file 10020_2024_834_MOESM2_ESM.tif]

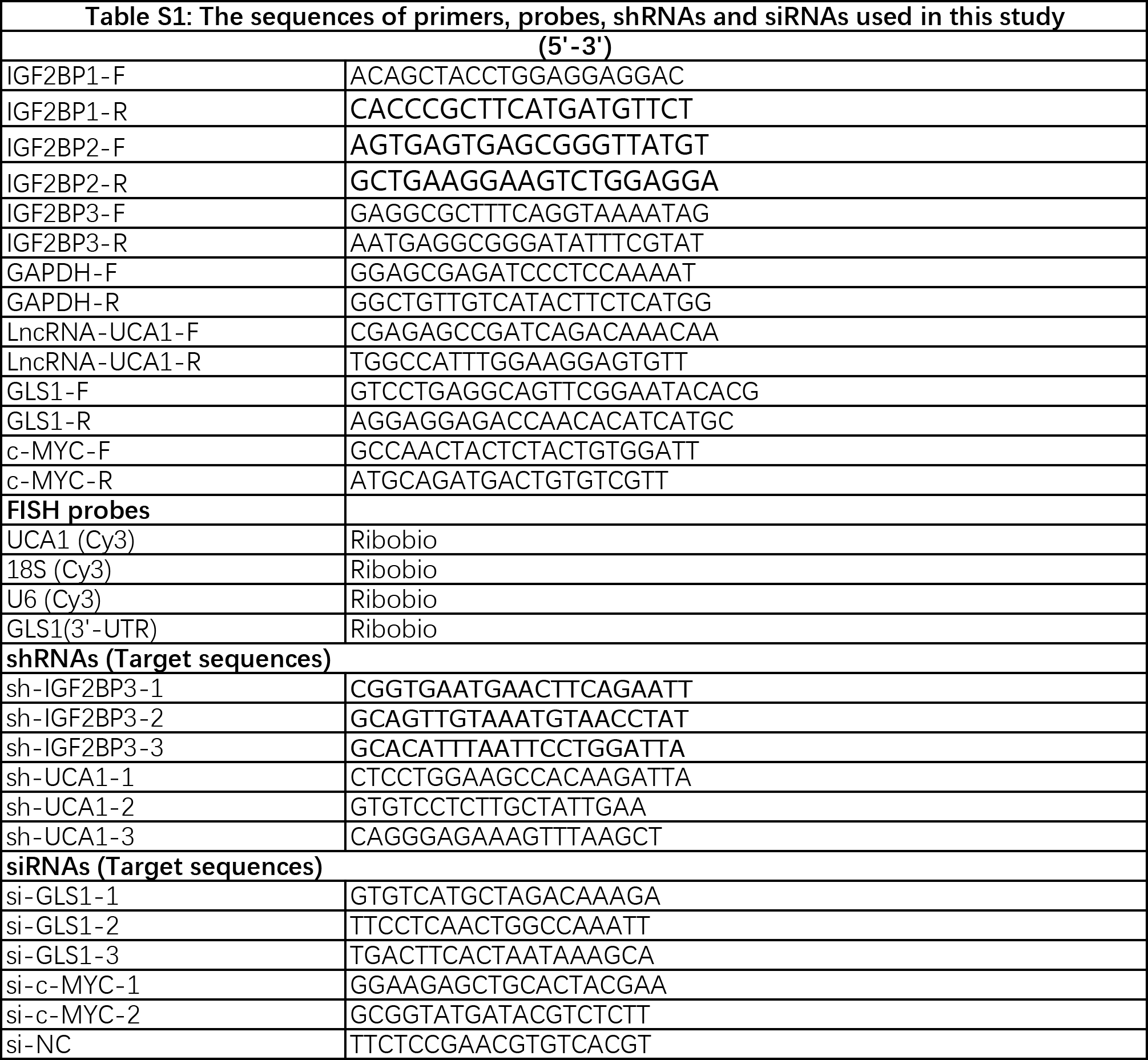

Supplement: Supplementary file 3 — Supplementary Material 3. [file 10020_2024_834_MOESM3_ESM.tif]

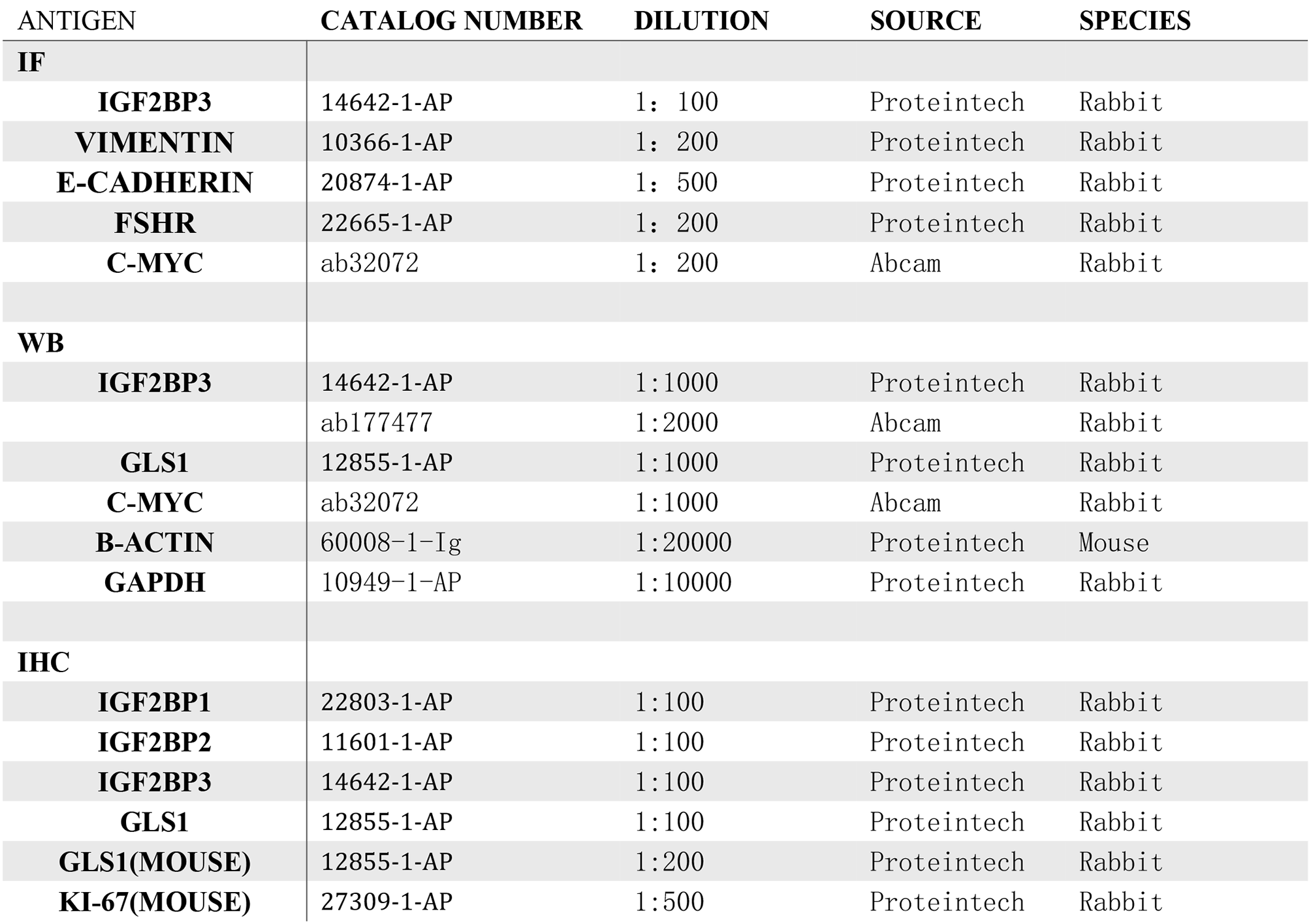

Supplement: Supplementary file 4 — Supplementary Material 4. [file 10020_2024_834_MOESM4_ESM.tif]
